# Supplementary figures and images for: Distinct behavior of the little finger during the vertical translation of an unsteady thumb platform while grasping
Source: Sci Rep. 2021 Oct 26;11:21064. doi: 10.1038/s41598-021-00420-5 (PMC8548443; doi:10.1038/s41598-021-00420-5)

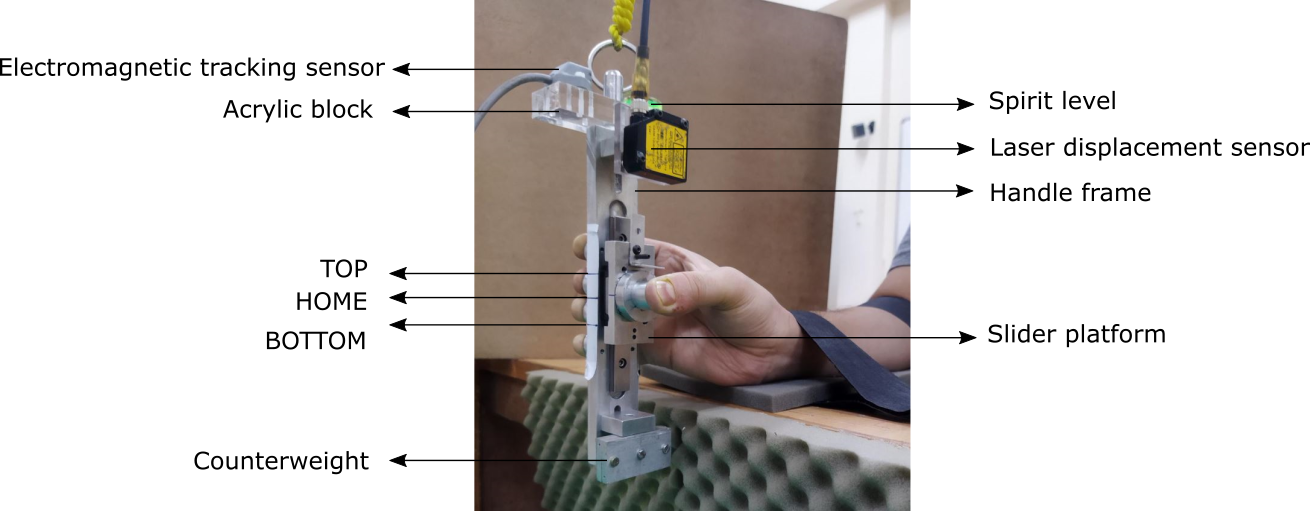

Supplement: Supplementary file 1 — Supplementary Figure S1. [file 41598_2021_420_MOESM1_ESM.tiff]

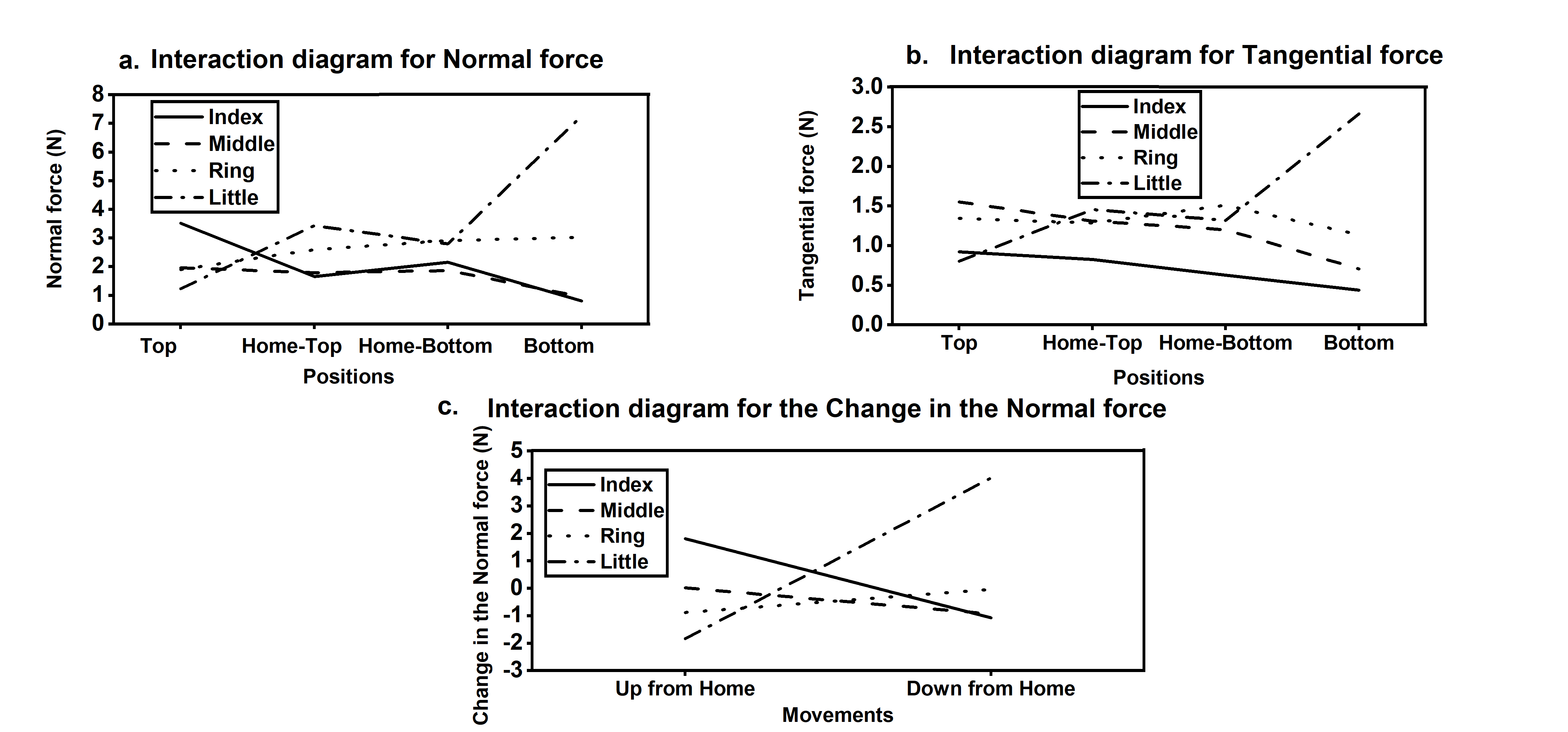

Supplement: Supplementary file 4 — Supplementary Figure S4. [file 41598_2021_420_MOESM4_ESM.tif]
