# Supplementary material for: Distinct behavior of the little finger during the vertical translation of an unsteady thumb platform while grasping
Source: Sci Rep. 2021 Oct 26;11:21064. doi: 10.1038/s41598-021-00420-5 (PMC8548443; doi:10.1038/s41598-021-00420-5)
Supplement: Supplementary file 5 — Supplementary Legends. [file 41598_2021_420_MOESM5_ESM.docx]

**Distinct behavior of the little finger during the vertical translation of an unsteady thumb platform while grasping**

**Banuvathy Rajakumar^1^** and **Varadhan SKM^2*^**

^1,2^Department of Applied Mechanics, Indian Institute of Technology Madras, Chennai, India.

**Corresponding Author:**

Dr Varadhan SKM

Associate professor

Department of Applied Mechanics

Indian Institute of Technology Madras

Chennai - 600036

Tamil Nadu

+91-44-22574071

Email address: skm@iitm.ac.in

Corresponding Author ORCID: 0000-0002-5746-2340

**SUPPLEMENTARY INFORMATION FILE**

**1. Supplementary Figure S1** Closeup labelled photograph of the participant holding the handle

**2. Supplementary Video S2** Video of a participant performing a trial in trapezoid condition.

**3. Supplementary Video S3** Video of a participant performing a trial in inverted trapezoid condition.

**4. Supplementary Figure S4 Interaction diagrams (**a). Interaction diagram for Normal force shows that the little finger normal force (7.25N) when the thumb was at BOTTOM position was significantly (p<0.001) greater than the index finger normal force (3.51N) when the thumb was at the TOP position, (b). Interaction diagram for Tangential force shows that the little finger tangential force (2.66N) when the thumb was at BOTTOM position was significantly (p<0.001) greater than the index finger tangential force (0.92N) when the thumb was at the TOP position, (c). Interaction diagram for the Change in the normal force shows that the change in the little finger normal force (4.01N) when the thumb was moved to the BOTTOM was significantly (p<0.001) greater than the change in the index finger normal force (1.79N) when the thumb was moved to the TOP from HOME.
